# Managing the spread of disease with mobile phone data

**DOI:** 10.1016/j.jdeveco.2020.102559

**Published:** 2020-11

**Authors:** Sveta Milusheva

**Affiliations:** The World Bank, USA

**Keywords:** Health, Big data, Epidemics, Mobility, Public policy, Mobile phones

## Abstract

While human mobility has important benefits for economic growth, it can generate negative externalities. This paper studies the effect of mobility on the spread of disease in a low-incidence setting when people do not internalize their risks to others. Using malaria as a case study and 15 billion mobile phone records across nine million SIM cards, this paper quantifies the relationship between travel and the spread of disease. The estimates indicate that an infected traveler contributes to 1.66 additional cases reported in the health facility at the traveler's destination. This paper develops a simulation-based policy tool that uses mobile phone data to inform strategic targeting of travelers based on their origins and destinations. The simulations suggest that targeting informed by mobile phone data could reduce the caseload by 50 percent more than current strategies that rely only on previous incidence.

## Highlights

• Anonymous telecom data is used to quantify effect of travelers from high to low disease incidence areas on infection rates.
• The paper demonstrates importance of travel for malaria incidence in low-incidence areas in Senegal.
• Mobile phone data can be used to develop more cost-effective strategies for targeting travelers that can spread disease.
• The methods can be applied to study spread of other diseases such as tuberculosis or COVID-19.

## 1 Introduction

Increasing domestic and international mobility has magnified the devastating consequences of infectious diseases: more than 11,000 deaths from Ebola, 440,000–1,300,000 cases of Zika infections in 13 countries, and most recently, over 21.2 million infections and 761,000 deaths from COVID-19^1^ across 188 countries and territories (Bogoch et al., 2016; WHO, 2020a; WHO, 2020b). Negative externalities from mobility are also relevant for long-standing diseases that society is working to eliminate. For example, Venezuela, the first country certified by the World Health Organization (WHO) for eliminating malaria in its most populated areas in 1961, experienced a dramatic resurgence of the disease in 2016, in part due to migrant workers in the mining region becoming sick, traveling home, and spreading the disease to their home villages and cities (Casey, 2016). This paper uses a case study of malaria in Senegal to demonstrate how to harness big data to estimate the size of this externality of movement and apply the results toward more effective policy targeting. The method can be applied more broadly to inform policies related to mitigating the spread of infectious diseases.

The economics literature identifies malaria eradication as having important impacts on adult income and consumption (Bleakley, 2010; Cutler et al., 2010; Venkataramani, 2012), real estate wealth (Hong, 2011), long term health including chronic disease and disability (Hong, 2013), and test scores and educational attainment (Barofsky et al., 2015; Lucas, 2010; Venkataramani, 2012).^2^ Ninety-nine countries have been certified by the WHO as malaria free; however, Sub-Saharan Africa, which accounted for 93 percent of all malaria deaths in 2018, has only had a single successful case of elimination (WHO, 2019). Previous work has studied malaria prevention/treatment in short-term settings, focusing on the pricing of malaria interventions (Cohen and Dupas, 2010; Cohen et al., 2015; Dupas, 2014; Laxminarayan et al., 2010; Tarozzi et al., 2014) and the adoption of preventative or antimalarial treatment (Adhvaryu, 2014; Apouey et al., 2018; Armand et al., 2017). There has been limited economics research examining the persistence of the disease and factors related to maintaining areas disease-free once elimination is accomplished in the short-term.

This paper uses novel data to estimate the role of a constraining factor for elimination: the reintroduction of malaria into elimination zones by population movement. Although this phenomenon has been documented in at least 61 countries by the epidemiology literature, the size of the effect has not been measured in a rigorous framework that controls for other factors (Cohen et al., 2012; Lu et al., 2014; Tatem et al. (2017)). In this research, I quantify the negative externality of mobility empirically using a pre-elimination setting in Senegal. By measuring the incidence that can be attributed to population movement, I generate predicted values that allow for simulation of different targeting strategies. This paper shows how aggregated big data on individuals’ geolocations can inform more cost-effective targeting strategies to reduce transmission generated by population mobility. Such a strategy could be a complementary component of any campaign to eliminate malaria.

The main challenge in estimating the size of the externality from mobility is that although disease transmission may respond quickly to changes in migration patterns, existing survey data that record these patterns are often infrequent or do not have coverage across a country.^3^ Therefore, the only strategy available for policy makers to address this externality is using incidence in the previous year to identify where and who to target. This paper significantly improves on this strategy by utilizing mobile phone data for 9.5 million SIM cards in Senegal in 2013. The analysis extracts patterns of movement between different areas from the approximate locations of 15 billion calls and texts. For each month and health facility area, I measure the number of incoming travelers from other regions weighted by the incidence of malaria in these regions and the length of time spent in the origin and destination to calculate “expected imported malaria cases.” I study an area of Senegal that is close to elimination of malaria, to focus on reintroduction effects.

I estimate the impact of imported incidence on total malaria incidence in this low-malaria setting using a linear dynamic panel data model and controlling for time fixed effects. If infected travelers only lead to the displacement of a malaria case from the origin to the destination without generating any externality, then a standard model would predict for each expected imported case one additional case reported in the destination. Instead, I find that one additional expected imported case in a low-malaria area leads to 1.094 cases of malaria reported in the current month and 0.563 cases reported in the following month, for a total impact of 1.66 cases. Using rainfall proxies and month fixed effects, I control for seasons and holidays, which drive a large amount of movement in Senegal and are correlated with malaria incidence. I also show that mobility alone is not correlated with malaria, and incidence in the origin is not correlated with incidence in the destination. The combination of mobility, incidence in the origin, and duration is required for the accurate measurement of the impact of travel and for the predictive modeling used for targeting simulations.

The negative externality from travel is generated when people are unaware of their risk to others because they do not know that they are disease vectors.^4^ Given the benefits of travel, people are unlikely to internalize the externality and choose not to travel to prevent infecting others. Policy makers then face trade-offs between economic growth and improving public health in designing policies to reduce travel-linked malaria cases. This paper provides a useful framework for strategic targeting of high-risk populations in low-incidence areas to reduce negative externalities from travel. The paper considers two categories of targeting: (1) high-risk travelers entering a low-malaria region from a high-malaria region and (2) all travelers in only specific areas of low-malaria regions that are likely to receive many high-risk travelers.^5^ Within each category, I compare a strategy that incorporates telecom information on travelers’ origins and destinations with a strategy that only uses historical information on incidence. Using the budget allocated in Senegal for these types of activities ($400,000), I demonstrate that the cost-effective strategy using mobile phone data performs over 50 percent better compared with the strategy that relies only on incidence in the previous year.

This paper uses high-frequency and spatially disaggregated data on mobility and disease to improve the accuracy of the predicted impact and build on previous health literature that has established travel as a risk factor for contracting malaria (Montalvo and Reynal-Querol, 2007; Lynch et al., 2015; Osorio et al., 2004; Siri et al., 2010; Littrell et al., 2013). While recent research in this area has begun to use mobile phone data to obtain a measure of mobility at a high frequency, few papers have looked at both mobility and disease dynamically across time (see Lai et al. (2019) for a review of the literature and Oliver et al. (2020) for a policy-focused review, targeted at the COVID-19 pandemic).

Several papers produce a static analysis of the relationship between mobility and disease, identifying sources and sinks of the disease without factoring in seasonal changes in mobility and disease prevalence (Wesolowski et al., 2012; Chang et al., 2018; Ihantamalala, 2018; Tatem et al., 2009; Le Menach, 2011; Ruktanonchai et al., 2016). Seasonal variation in biological factors and seasonal population movements are important for many infectious diseases, and failing to account for seasonality could lead to misallocation of resources (Buckee et al., 2017).

Some papers leverage the high-frequency nature of mobile phone data but do not combine it with incidence data. Wesolowski et al. (2017) look at seasonality of movement patterns across Kenya, Pakistan, and Namibia but connect it to disease only theoretically. Similarly, Tompkins and McCreesh (2016) use a limited mobile phone data set from Senegal to study patterns in mobility over time.^6^ They discuss the theoretical impacts on malaria but do not combine their analysis with disease data.

Two papers combine high-frequency mobile phone data and high-frequency disease incidence data. Wesolowski et al. (2015a) conduct this analysis for dengue, but not in a regression framework. Therefore, they do not measure the size of the impact of travel compared with other factors and cannot control for location-specific or time-specific unobservables. Wesolowski et al. (2015b) use a regression framework for rubella, but they do not control for time fixed effects. This could bias the estimates if both rubella and travel are influenced by other factors that are also correlated with time. This paper contributes to the existing work by aiming to produce a less biased estimate of the effect of imported malaria, using a linear dynamic panel data model and controlling for time fixed effects. This makes it possible to quantify the size of the contribution of travel to malaria incidence, which enables cost-benefit simulations of targeting policies.

Although the paper focuses on malaria elimination, it has implications for other diseases whose spread has been associated with travel (Adda, 2016; Oster, 2012; Prothero, 1977; Balcan et al., 2009; Stuckler et al., 2011; Tam et al., 2016). If cell phone data are obtained for other countries or for different diseases, it is possible to replicate the analysis using the methods developed in this paper and a modified model for the specific epidemiology of the disease. The data can also be used to measure the effectiveness of policies targeted at mobility, as was done for the case of Ebola in 2014 (Peak et al., 2018). Even if cell phone data is not available continuously, it is possible to use predictions of mobility based on a model calibrated with limited cell phone data to study the spread of a disease (Milusheva, 2020). I demonstrate how new sources of big data can be used to measure externalities associated with travel to develop more effective targeting strategies. This further expands the use of big data for development in economics in areas such as risk-sharing (Blumenstock et al., 2016), measuring poverty (Blumenstock et al., 2015; Blumenstock, 2016), and providing credit to the poor (Björkegren and Grissen, 2019).

The paper begins in section 2 by providing some background and describing the data. Section 3 models the relationship between malaria and population movement. Section 4 outlines the empirical results linking travel to malaria and provides robustness checks. Section 5 examines the cost-effectiveness of various targeting policies, and section 6 concludes.

## 2 Background and data

### 2.1 Characteristics of malaria

Malaria is an infectious disease that requires two hosts–humans and mosquitoes–to spread. The malaria cycle for *P. falciparum*, the parasite causing 100 percent of cases in Senegal, can take several weeks (WHO, 2014). After an infected individual is bitten by a mosquito, there is an incubation period lasting around nine days within the mosquito (Killeen et al., 2006).^7^ If the mosquito survives the incubation period, it can bite and infect a healthy individual, after which there is a second incubation period within the human of around 15 days (Smith and McKenzie, 2004; Hoshen and Morse, 2004). Symptoms will appear at the end of this period and the individual will become infectious.^8^ Combining the two incubation periods, a secondary case will take around one month to appear after a primary case.^9^ Untreated malaria can have a duration of around 200 days, but within the first three days of treatment with artemisinin-based combination treatments, the majority of parasites are eliminated (Nosten and White, 2007).

This paper focuses on the role of human behavior in the spread of the disease.^10^ Population movement can lead to the spread of malaria in low-malaria zones through two channels. The first is residents of these zones who travel to high-malaria areas and become infected when infected mosquitoes bite them. The second channel is visitors or migrants who live in a high-malaria area and travel to a low-malaria area. Since the symptoms of malaria do not appear for around two weeks, residents and visitors can travel feeling healthy. Once at the new location, the person can become symptomatic as well as infect mosquitoes. These infected mosquitoes can infect other individuals and pass on the disease.

### 2.2 Health system and malaria in Senegal

Senegal is geographically divided into 14 health regions and 76 health districts. The main points of service for malaria cases are the 1247 health posts in the country. In addition, rural health huts and community health workers provide care for those living far from a health post and report the cases to the closest health post.

Since the establishment of the National Malaria Control Program (PNLP) in 1995, the program has coordinated a variety of measures and policies that have led to a reduction in deaths attributed to malaria, from 12.93 per 100,000 people in 2000 to 8.26 in 2013 (PNLP INFORM and LSHTM, 2015). Currently, the north of the country has very low incidence of malaria and is at the level considered ready for elimination by the WHO (one case per 1000 people, which is known as the pre-elimination phase). In contrast, the south still has a high case load, with some districts having as many as 270 cases per 1000 people.^11^ The mosquitoes required to spread the disease are present across the country, but certain environmental factors, such as the length of the rainy season, lead to heterogeneity (Ndiath et al., 2012). The Government of Senegal is striving to continue reducing the case load in the south while aiming to eliminate it completely in the north. However, infected individuals traveling from the south to the north may hinder elimination efforts in the north.

Low-incidence areas that are close to elimination may experience the largest externality from population mobility for three key reasons: (1) without these travelers, the disease could be reduced to zero and require lower government expenditures; (2) immunity to the disease only exists in high-malaria areas; therefore, a traveler entering a high-malaria area is less likely to lead to a new infection even if he or she infects additional mosquitoes in the area; and (3) the infection in a low-malaria area is likely to be more severe due to the lack of exposure to the disease. Therefore, I focus on parts of Senegal that are at the pre-elimination stage.

**Fig. 1:**
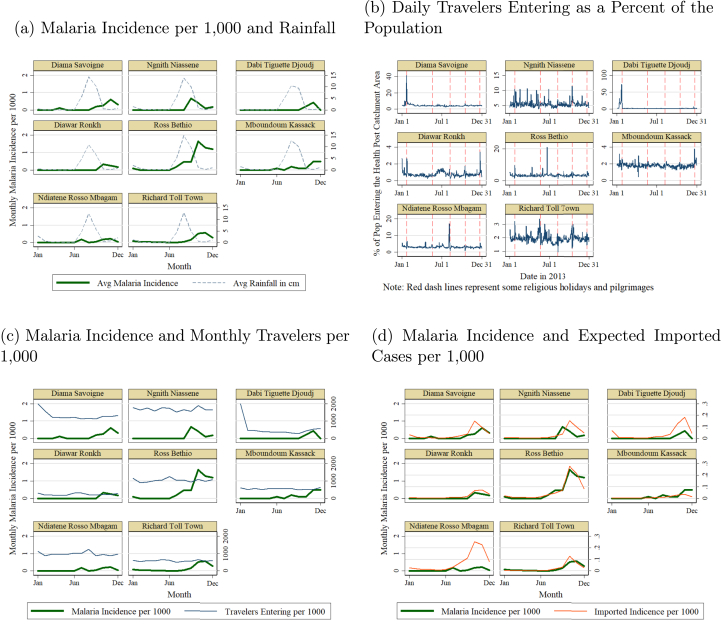
Malaria Incidence, Rainfall, and Population Mobility: Example of Health Post Areas in Richard Toll District. *Notes*: Panels show the values for each health post catchment area in Richard Toll. The y scale is rescaled for panel (b) to better illustrate the movement patterns in each health post area, given the large outliers in Diama Savoigne and Dabi Tiguette Djoudj. The scale in panel (c) is not rescaled to allow for comparison across health facility areas, but it is bounded at 2,000 (for Diama Savoigne and Dabi Tiguette Djoudj, the value in January goes up to around 4,000).

**Fig. A.2:**
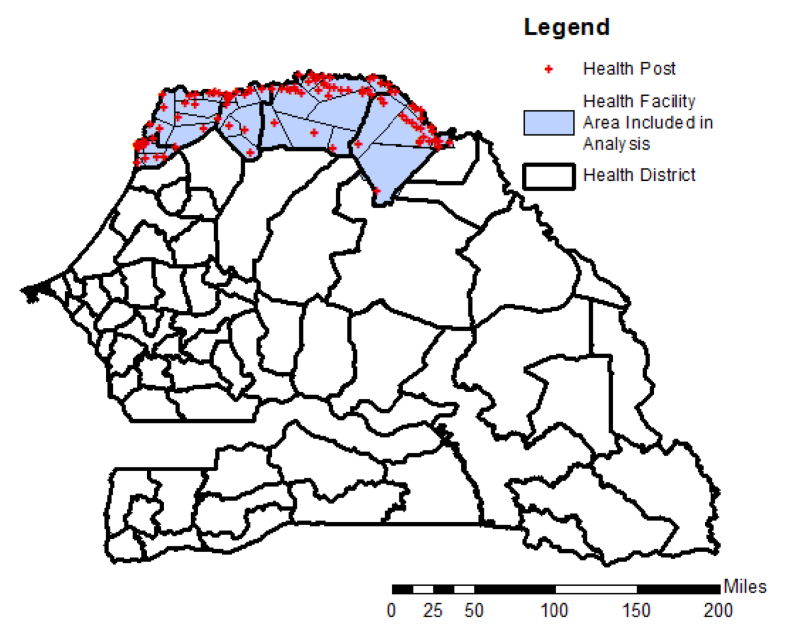
Senegal Health Districts and Location of Health Posts in the North Used in the Analysis. *Notes*: The five very low-malaria health districts used in the analysis are subdivided into health post catchment areas that group health posts and mobile phone towers together.

**Fig. A.3:**
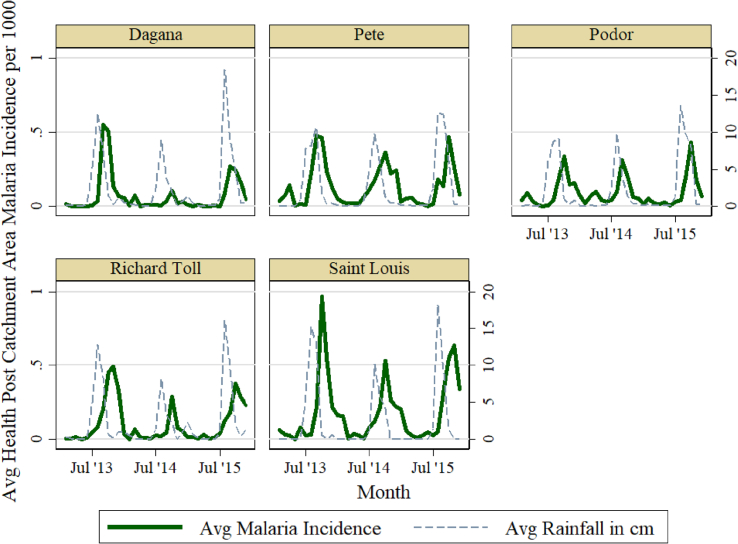
Average Monthly Health Post Catchment Area Malaria Incidence per 1000 Population and Average Monthly Rainfall, January 2013–December 2015, by Health District.

I study five districts that have among the lowest rates of malaria incidence. Data are disaggregated at the health post level and available for all 117 health posts in these districts. Malaria data are not available at this high spatial resolution for any other districts. I group health posts in close proximity to form 36 health post catchment areas (see Figure A.2 in Appendix A). Incidence is based on data collected from each health post on all new cases in the reporting month. I use incidence data at the district level for the country to calculate imported incidence.^12^ On average, the health post areas in the five low-malaria districts have around 0.1 case per 1000 people per month (see Figure A.3 in Appendix A for average monthly incidence). Fig. 1, panel a, uses one health district to illustrate monthly malaria incidence per 1000 people at the health post catchment area level. The figure overlays the monthly cumulative rainfall in centimeters.^13^ The comparison of cases and rainfall demonstrates the seasonality of malaria in Senegal and the relationship between rainfall and malaria. See Figure A.3 for a longer time trend that shows how the peak number of cases occur one to two months after the peak in rainfall.

Three main challenges arise with using clinical data: incomplete data reporting, presumptive diagnosis based on symptoms rather than testing, and non-utilization of the public health system (Alegana et al., 2013). In the data, only 12 of 1416 health post-month observations are missing for 2013. In addition, 99 percent of suspected cases were tested parasitologically using a rapid diagnostic test (RDT) in the five districts analyzed. Since total and imported malaria cases are calculated based on case data, health facility utilization should not bias the estimates as long as it is relatively uniform across the country. Based on the Demographic and Health Survey (DHS) data for all the regions, a health facility was visited for fever in children under age five years in 46 percent (standard deviation 6.5 percentage points) of the cases (ANSD and ICF International, 2015). I assume uniform utilization in the main analysis, and I include a robustness check where cases are scaled by regional utilization in the DHS.

### 2.3 Population movement and data in Senegal

Senegal has large flows of long-term and permanent migration, with 27 percent of the population recorded as internal migrants in 2004 (Fall et al., 2010).^14^ A large part of this migration is rural to urban (Fall et al., 2010; Goldsmith et al., 2004). In turn, long-term migration can lead to commuting patterns, as people return home to visit family and friends or receive visitors from home (Cho et al., 2011). Fall (1998) finds that 87 percent of male and 81 percent of female migrants in Dakar visited their home areas, with the majority of visits occurring for holidays, family ceremonies, and religious festivals.

Circular migration plays an important role, with one study finding over 80 percent of unmarried Jola youth traveling to the cities in October and then coming back before the rice harvest in June–July (Linares, 2003). Along with rice, the Senegal River Valley region engages in horticulture such as onion and tomato production. This brings a large number of seasonal workers, especially for the tomato export sector (Maertens et al., 2012). Agricultural production also leads to trade and a large number of traders and trucks coming to markets in these areas after the harvest seasons. Additionally, there are still pastoral groups that travel within a set territory (Adriansen, 2008).

The data used to measure short-term movement come from phone records made available by Sonatel and Orange in the context of the second phase of the Data for Development Challenge (Montjoye et al., 2014). The data consist of 15 billion call and text records for Senegal between January 1, 2013 and December 31, 2013 for all of Sonatel's user base.^15^ The data contain information on all calls and texts made or received by a SIM card, their time, date, and location of the closest cell phone tower. These variables enable tracking the SIMs in space as they make calls from different tower locations. The data are anonymized, with a random ID provided that makes it possible to track the same SIM over time, but there is no identifying information on the individuals. On average, there are 1657 calls or texts per ID during the year and an ID has a call or text on 155 days.

Each tower is assigned a health district based on its GPS coordinates. I follow previous literature to assign individuals a daily health district based on the cell tower of the last call or text of the day (Ruktanonchai et al., 2016). For days with no calls, I replicate the procedure in Wesolowski et al. (2012) and assign the health district of the day closest to the one missing.^16^ A health district is assigned to each SIM for every day of the year.

Movement is defined as a change in location from one health district to another between two consecutive days. An overnight stay is necessary for someone to be infected because the mosquito that spreads malaria is only active at night. Therefore, movements, such as travel to markets, that do not require an overnight stay are unlikely to lead to spread of malaria. For other diseases, it may be necessary to adjust how mobility is measured. For diseases like influenza or COVID-19, more frequent measures of movement are necessary to capture the epidemiology of the disease. The high-frequency nature of mobile phone data makes it flexible for measuring movement at the appropriate level for a particular disease.^17^

Over 80 percent of all Sonatel SIM cards take at least one trip and more than a quarter million travel on average each day. On average annually per SIM, there are 10 trips to almost five health districts. In addition to assigning the towers within the study area to a health district, I assign them to a health post catchment area based on their GPS location. Therefore, each traveler entering one of the five health districts is assigned a specific health post catchment area based on the last call or text of the day.^18^

Fig. 1, panel b, shows the number of people entering a health post catchment area each day as a percentage of the population in that catchment area for an example district. On average for all the health post catchment areas, around 3 percent of the population of that area enters on any given day. The movement is aggregated at the monthly level to match the malaria case data. Even at the monthly level, the percentage of people entering varies widely by health post catchment area within a district (Fig. 1, panel c). For health post catchment areas where an important religious leader resides, on certain religious holidays the number of people entering is close to or over 50 percent of the population of the area. For other health posts, the beginning of certain agricultural seasons or the end of the season and increased trade of agricultural products lead to large jumps in people entering. This variation makes it possible to study the impact of people entering on malaria cases in these areas, which are geographically close together and otherwise very similar. Importantly, not all movement is equally likely to spread malaria. High travel during the months when incidence is close to zero across the country will not have an impact. This is shown in Fig. 1, panel c, which also includes malaria incidence in the health facility areas along with population mobility.

In 2013, Sonatel had slightly more than 9.5 million unique phone numbers on its network while the population of Senegal was 13.5 million.Two sets of people are potentially excluded from the data and need to be accounted for–those without a phone and those with a phone but using a different mobile provider. Based on the Listening to Senegal Survey (LSS) done in 2014, 12.3 percent of adults age 18 and over never use a mobile phone. Sonatel is one of three mobile phone providers. Based on the LSS, Sonatel is the main provider for 80 percent of those surveyed with a cell phone, and 88 percent of those with a cell phone have a Sonatel SIM card (ANSD, 2014a). Therefore, only around 12 percent of adults with a SIM are excluded. Combining the two types of missing adults, 77.2 percent of adults are represented in these data.

I conduct checks to see how representative the data are for the two types of users that are not included—those without a SIM and those with a SIM from a different provider. I use the DHS survey to compare mobility patterns between women with and without a cell phone in the household.^19^ There is no statistical difference in whether a trip longer than a month was taken between those with and without a cell phone. Women with a cell phone in the household have a slightly higher average number of trips taken in the last year. Using the LSS, I compare several indicators between people who have a Sonatel SIM card and those who only have a SIM card from another provider. Based on t-tests, there is no significant difference between these categories of individuals in type of primary activity (p-value = 0.344), sector of primary activity (p-value = 0.863), source of drinking water (p-value = 0.868), non-food expenditures over the past month (p-value = 0.193), and non-food expenditures over the past 12 months (p-value = 0.115). The third missing group is children. If children travel, they are likely traveling with adults (although they might travel less on average if they do not always travel every time an adult travels). Since there are no data comparing the short-term movements of adults and children in Senegal, I use a uniform weight of 1.4 to represent the full population and get an upper bound on movement. The weighted data likely overestimate total movement and underestimate the impact of each trip. Unweighted results are provided in the robustness section (section 4.2).

One of the limitations of mobile phone data is that movement from abroad cannot be measured. In Senegal, 2 percent of the population is international migrants and 1.2 percent of the population emigrated from Senegal (ANSD, 2014b). In 2013, only 0.23 percent of the population entered Senegal.

## 3 Empirical model

The empirical specification is derived from a model of malaria that is based on previous models used in Smith and Ellis McKenzie (2004), Cosner et al. (2009), and Torres-Sorando and Rodriguez (1997). Four key assumptions allow me to simplify the model so that incidence in the current month is dependent on incidence in the last month using a linear functional form. Expected imported cases enter as a linear additive term as in Torres-Sorando and Rodriguez (1997). The model, assumptions, and implications are described in detail in Appendix B. I start out estimating equation (1) using ordinary least squares, with imported incidence calculated using equation (2):

(1) $$\begin{array}{ccc} x_{it} & = & \beta_{1}x_{it-1}+\beta_{2}E\left(I_{it}\right)+\alpha Z_{it}+\gamma_{i}+\delta_{t}+\varepsilon_{it} \end{array}$$

(2) $$\begin{array}{ccc} E\left(I_{it}\right) & = & \frac{1}{H_{it}}\sum_{j\neq i}\sum_{p_{t}\in j}T_{ip}\left(x_{jt}T_{jp}\right) \end{array}$$

In this model, *x*_*it*_ represents the incidence, or number of people infected in location i at time t per 1000 people in location i.^20^ Location i is one of the 36 health post catchment areas and t is the month. The *β*_1_ parameter estimates the average number of new cases per 1000 generated in the following month from cases in the current month. In contrast to epidemiological models, where this parameter is estimated separately for each area, I estimate an average effect across locations and time to include health post area fixed effects, *γ*_*i*_, and month fixed effects, *δ*_*t*_. This allows me to control for malaria seasonality and unobservable characteristics of a health post area that might bias the estimate.

I use the mobile phone data to calculate expected imported malaria cases entering and divide them by the population of the area they enter, *H*_*it*_, to calculate the expected imported incidence, $E\left(I_{it}\right)$. The probability of an infected case entering depends on the origin *j* of a person, *p*_*t*_, how long the person spent there, and the length of time in the destination, *i*. On average, people spent 9.9 days in the origin before traveling and 10.5 days in the destination, although in both cases the median is 2.^21^ I make two assertions:

1. The likelihood that a person, *p*_*t*_, is infected is based on the fraction of the month up to 15 days spent in the origin district j, *T*_*jp*_, and the monthly incidence rate in j, *x*_*jt*_. The timing is cut off at 15 days because the incubation period is 15 days.^22^
2. The contribution of an imported case to a new location is calculated as a fraction of time up to 15 days spent in the destination location i, *T*_*ip*_. Based on survey data on all cases in Richard Toll in 2013, on average people visit a health facility around six days after exhibiting symptoms, and 90 percent visit by 12 days after symptoms occur. Those who test positive for malaria (which is all cases in the data I use) are provided free antimalarial treatment, which means that within three days the level of parasites is significantly reduced and they can no longer infect mosquitoes. Therefore, there is a period of at most 15 days during which people are infectious and contributing to additional cases in this data.

Agricultural seasons can lead to increased mobility due to seasonal workers and increased trade. Agricultural seasons are also correlated with rainfall, which could affect mobility via the conditions for travel (quality of roads), and rainfall is also highly correlated with malaria incidence. I control for this potential confounder by including rainfall covariates in the specification (*Z*_*it*_ includes zero, one, and two lags of rainfall).^23^ *ε*_*it*_ represents idiosyncratic shocks. I use bootstrap standard errors clustered at the health post catchment area level given the estimated nature of the imported incidence variable and that errors are likely correlated within panels.^24^ The main coefficients of interest are *β*_1_ and *β*_2_, which represent the number of secondary cases generated by infected travelers and the number of primary malaria cases imported by infected travelers.

The dynamic panel model with a relatively short panel of 12 time periods could introduce bias if the error term is mechanically correlated with the lagged dependent variable on the right-hand side (Nickell, 1981). I study this by comparing the fixed effects model with a random effects model. Given the results of this comparison, the preferred specification used is an augmented version of the Arellano-Bond generalized method of moments (GMM) estimator, the Arellano-Bover/Blundell-Bond estimator, which was designed to efficiently address situations with “small T, large N” panels (Arellano and Bond, 1991; Arellano and Bover, 1995; Blundell and Bond, 1998). An important identifying assumption for the estimator is that there is no serial correlation except first-order serial correlation in differences. I include an Arellano-Bond test for autocorrelation to ensure this assumption holds.

## 4 Results

### 4.1 Quantifying the effect of imported cases

Each imported case of malaria is associated with 1.23 cases of malaria in the current period and 0.330 cases in the next period, based on the fixed effects model (Table 1, column 1). This specification assumes the externality from locally generated and imported cases will be the same. I explicitly test this by including lagged imported incidence along with lagged local incidence (column 2). The coefficient on lagged imported incidence is not significantly different from the coefficient on lagged local incidence, which implies that there is no differential effect between lagged imported and lagged local incidence. Therefore, the coefficient on lagged total incidence provides a measure of the secondary cases generated by imported cases. I estimate a random effects model to test if there could be a dynamic panel bias due to the inclusion of fixed effects with a relatively short panel (column 3). I control for several time-invariant characteristics of the health facility areas, including population density, a dummy for urban areas, and a dummy for health facility areas that are not along the border of the country.

**Table 1:** Effect of imported malaria incidence.

	(1)	(2)	(3)	(4)
	Fixed Effects	Fixed Effects	Random Effects	Arellano Bond
Imported Incidence	1.230∗∗ (0.535)	1.175∗ (0.566)	0.864∗ (0.458)	1.094∗∗∗ (0.451)
Lag Incidence	0.330∗∗∗ (0.0543)		0.418∗∗∗ (0.0597)	0.563∗∗∗ (0.126)
Lag Imported Incidence		0.449 (0.301)		
Lag Non-Imported Incidence		0.340∗∗∗ (0.0623)		
Rain in cm	0.00639 (0.00975)	0.00592 (0.0105)	0.00449 (0.00898)	−0.000654 (0.00844)
Lag Rain in cm	0.0292 (0.0196)	0.0294 (0.0195)	0.0300∗ (0.0184)	0.0269 (0.0193)
Lag 2 Rain in cm	0.0381∗∗ (0.0165)	0.0372∗∗ (0.0163)	0.0382∗∗∗ (0.0149)	0.0319∗∗ (0.0169)
Month FE	Yes	Yes	Yes	Yes
Health Post x Month Obs	432	396	432	432
R-squared	0.509	0.512		
Hausman Test Comparing Column 1 and Column 3			p-value = 0.0083	
Arellano-Bond test for AR(1)			p-value = 0.001	
Arellano-Bond test for AR(2)			p-value = 0.245	
Testing Predictions				
Test Lag Imported = Lag Non-Imported (Column 2)			p-value = 0.491	
Test Imported = 1 (Column 4)			p-value = 0.798	
Test Lag Incidence = 0.273 (Column 4)			p-value = 0.0404	
Test Imported + Lag Incidence = 1 (Column 4)			p-value = 0.046	

Cluster bootstrap standard errors in parentheses.

∗∗∗ p < 0.01, ∗∗ p < 0.05, ∗ p < 0.1.

*Notes:* Column 3 includes controls for health post area population density, a dummy for urban health post areas, and a dummy for non-border health post areas.

A Hausman test comparing the two models finds they are significantly different. Given that each model has potential to be biased, since the fixed effects model might have some dynamic panel bias while the random effects model might have omitted variable bias, I use an augmented Arellano-Bond system GMM specification (Table 1, column 4). Based on this model, for each imported case of malaria per 1,000, there are 1.094 cases per 1000 reported. In addition, for each lagged case per 1,000, there is an additional 0.563 of a case generated the following month. This also represents the negative externality of an imported case the previous month. Therefore, the combined impact of an imported case on incidence in a low-malaria area is the sum of the impact in the current month and the following month, or 1.094 plus 0.563 cases, for a total of 1.657 cases per 1000. The Arellano-Bond AR(1) and AR(2) tests at the bottom of the table report the p-values for tests of first- and second-order serial correlation in the residual, providing support for the assumptions.

The epidemiological model on which the empirical specification is based leads to several testable predictions. The results are at the bottom of Table 1. The model implies that each imported case per capita contributes a case per capita to the incidence in the destination in the month of entry. With a p-value of 0.79 on the Wald test, the coefficient is not significantly different from 1. Additionally, based on the malaria model, the epidemiology literature would estimate *β*_1_ as 0.273.^25^ I find that the coefficient is significantly different and larger, implying that the parameters estimated by the epidemiological model would underestimate the externality. Finally, to measure whether there is an externality beyond a case being detected in one location versus another location due to travel, I test whether the sum of imported incidence and lag incidence equals 1. The sum is significantly different from 1 at the .05 level.

**Fig. 2:**
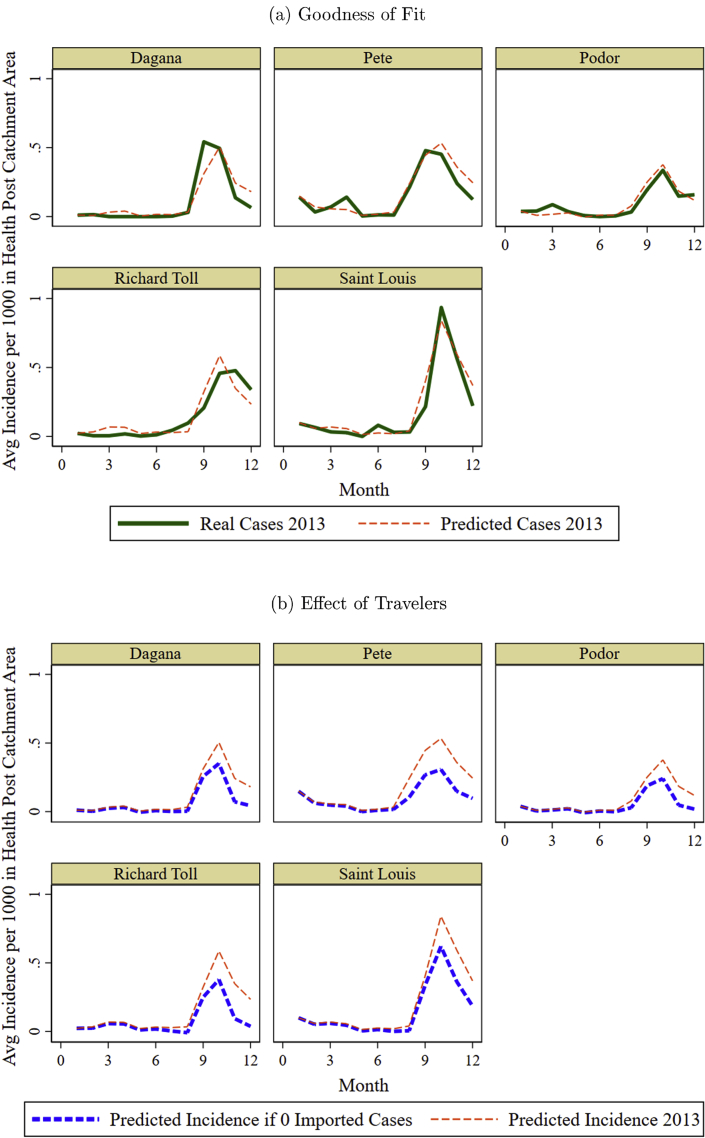
Predicted, Predicted without Imported Cases, and Actual Incidence Averaged Across Health Post Areas, by District. *Notes*: The thin dashed lines represent the monthly predicted malaria incidence averaged across health post areas within a district. This was calculated based on values for the parameters of the model drawn from their distributions. I conducted 500 replications and used the mean monthly incidence value per health post area. Panel a compares the predicted values with the actual malaria incidence, where the solid line is actual incidence averaged across health posts within a district. In panel b, the predicted incidence is compared with a scenario where no cases were imported by travelers, shown in thick dashed lines. Incidence with 0 imported cases was calculated using the same 500 replications for parameter values, but imported cases were set to 0.

I simulate a baseline scenario based on the augmented Arellano-Bond model and compare it with the actual data. I first bootstrap the data, generating 10,000 samples to estimate the distribution of the coefficients. I draw values from these distributions and calculate an incidence path for the year, replicating the procedure 500 times and then averaging paths across all the draws. In Fig. 2, panel a, I calculate average predicted incidence across health post areas by district and compare it with actual incidence. Similarly, I simulate incidence under the assumption that there is no imported incidence. Fig. 2, panel b, compares the results of this simulation with the baseline simulation. Imported incidence represents a substantial portion of the malaria incidence, especially for Richard Toll and Saint Louis. On average annually per health post, travel represents 41 percent of the incidence.

### 4.2 Mechanism evidence and robustness checks

**Fig. 3:**
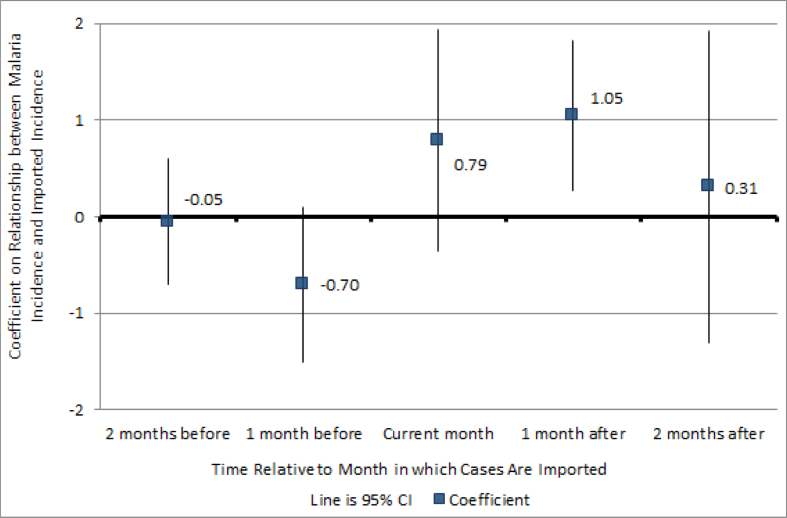
Estimated Impact of Future, Current, and Past Expected Imported Malaria Incidence. *Notes*: The figure was constructed based on a regression of current malaria incidence on imported incidence of malaria two months later, one month later, currently, last month, and two months ago, controlling for time and location fixed effects and rainfall covariates and clustering errors at the health post area level.

Fig. 3 shows the coefficients from a regression of malaria incidence on two lags and two leads of imported incidence, along with location and time fixed effects and rainfall controls. Malaria incidence two months earlier and one month earlier has no relationship with imported cases in the current period. Malaria incidence in the current period and one month later is associated with imported incidence, as both coefficients jump. Imported incidence does not seem to have a significant impact on incidence two months later.^26^ Since the sample size is much smaller after including the leads and lags, the standard errors are larger. Nevertheless, the trend in the size of the coefficient still demonstrates that future imported incidence does not drive current malaria incidence.

There are potentially other confounders for which I cannot control that may bias the relationship that I measure. I leverage the nature of how the imported incidence variable is created, to explore the existence of some of these confounders. The imported incidence variable combines two elements: the number of people traveling and the incidence of where they came from and how long they spent there (this is demonstrated visually in Fig. 1, panels c and d). There could be the same number of people going from *j* to *i* and from *k* to *m*, but if *j* has a high incidence while *k* has a low incidence, then *i* and *m* would experience different impacts. This enables me to test for some confounders by removing variation arising from one of the components of the variable and keeping only variation from the other component.

One source of confounders is unobservables that are correlated with mobility and malaria incidence. For example, as seen in the literature and in the mobility data, holidays increase population movement. Holidays may also independently impact malaria if, for example, people spend more time outside during the holidays, exposing themselves to mosquitoes. If this were the case, it would lead to an upward bias in the coefficient, an overestimate in the predictions on how much targeted interventions could help to reduce malaria, and incorrect cost-benefit calculations that overestimate the benefit. In contrast, trade is positively correlated with mobility and income. Therefore, there could be a scenario where higher trade and income lead to a higher likelihood of purchasing malaria preventative measures, which would decrease malaria incidence. This would lead to a downward bias in the coefficient and an underestimate of the impact of imported incidence.

I test for these confounders by regressing malaria incidence on total people entering (Table 2, column 2). There is no relationship between mobility and malaria incidence. There may be unobservables correlated with mobility during the malaria season and correlated with malaria. To test this, I scale mobility by average incidence across health districts in Senegal, rather than the incidence of the origin location. In effect, this weights movement during the malaria season much more than movement during other times. There is no relationship between this weighted variable of mobility and malaria incidence (Table 2, column 3).

**Table 2:** Decomposing imported incidence to test for confounders.

	(1)	(2)	(3)	(4)
	Baseline Model	Unscaled Travel	Travel Scaled by Avg Monthly Incid	Avg Travel Scaled by Monthly Incid
Travel/Imported Incidence	1.094∗∗∗ (0.451)	0.215 (0.311)	0.0290 (0.178)	0.00118 (0.137
Lag Incidence	0.563∗∗∗ (0.126)	0.564∗∗∗ (0.131)	0.588∗∗∗ (0.129)	0.588∗∗∗ (0.130)
Rain in cm	−0.000654 (0.00844)	−0.00468 (0.0130)	−0.00530 (0.00801)	−0.00538 (0.00795)
Lag Rain in cm	0.0269 (0.0193)	0.0329∗ (0.0235)	0.0290 (0.0192)	0.0288 (0.0189)
Lag 2 Rain in cm	0.0319∗∗ (0.0169)	0.0399∗∗ (0.0206)	0.0382∗∗ (0.0170)	0.0381∗∗ (0.0172)
Month FE	Yes	Yes	Yes	Yes
Health Post x Month Obs	432	432	432	432

Cluster bootstrap standard errors in parentheses.

∗∗∗ p < 0.01, ∗∗ p < 0.05, ∗ p < 0.1.

*Notes:* The augmented version of the Arellano-Bond GMM estimator is used in all specifications. Columns 2–4 show regressions where imported incidence has been constructed in an alternative way that does not incorporate the two needed elements: the incidence in the origin and the number of travelers. Column 2 shows the results from regressing malaria incidence on total travelers entering scaled by the population of the area entered without factoring in the incidence of the travelers' origins. In column 3, travelers are scaled by average monthly incidence in Senegal rather than incidence in their origin area. In column 4, an average value for travel between origins and destinations is calculated, and this average is scaled by the incidence of the origins.

Another possible source of confounders is if there are other relationships between origin and destination areas that may impact the incidence in both places in a particular way irrespective of travel. To rule this out, I calculate expected imported incidence based on the incidence of the origins but use the average number of travelers between origins and destinations rather than the actual number of travelers, in effect removing variation from mobility.^27^ There is no relationship between this variable and malaria incidence (Table 2, column 4). These tests are not able to rule out that there may be an unobservable that is correlated with both the combination of travelers and incidence in the origin and with incidence in the destination. Nevertheless, the tests help to rule out a large number of the confounders that we may be worried about, increasing confidence in the measurement of the coefficients.

I also conduct several additional placebo tests and robustness checks (Table 3). I test that the effect is only seen for malaria incidence and is not a reflection of a relationship between migrants and consultations at a health post. I use a variable for imported health caseload other than malaria. This variable is measured as the number of consultations at a health post, including all consultations with a nurse, all maternity consultations (excluding pre-natal), and all post-natal consultations. Any cases of malaria or fever are removed from the total, and the variable is scaled by the population of the health post area. This variable has no relationship with malaria incidence in the location where people enter (Table 3, column 2). I also use the original imported malaria incidence variable as the regressor, but I change the dependent variable to be non-malaria caseload as a proportion of the population, and I use lagged non-malaria caseload instead of lagged malaria incidence. Imported malaria incidence does not have a relationship with non-malaria caseload (column 3).

**Table 3:** Robustness checks.

	(1)	(2)	(3)	(4)	(5)
	Baseline Model	Travel Scaled by Non-Malaria Case Incid	Effect on Non-Malaria Case Incid	Unweighted	Scaled by Utilization
Imported Incidence	1.094∗∗∗ (0.451)	0.323 (0.337)	27.14 (28.707)	1.563∗∗∗ (0.509)	1.294∗∗∗ (0.449)
Lag Incidence	0.563∗∗∗ (0.126)	0.574∗∗∗ (0.144)	0.202∗ (0.121)	0.563∗∗∗ (0.129)	0.567∗∗∗ (0.128)
Rain in cm	−0.000654 (0.00844)	0.0444 (0.0560)	−0.605 (0.879)	−0.000654 (0.00790)	−0.00119 (0.0206)
Lag Rain in cm	0.0269 (0.0193)	0.0595 (0.0564)	−0.198 (0.677)	0.0269 (0.0181)	0.0703 (0.0466)
Lag 2 Rain in cm	0.0319∗∗ (0.0169)	0.0542 (0.0399)	−1.340∗∗ (0.586)	0.0319∗∗ (0.0156)	0.0812∗∗ (0.0398)
Health Post x Month Obs	432	432	432	432	432

Cluster bootstrap standard errors in parentheses.

∗∗∗ p < 0.01, ∗∗ p < 0.05, ∗ p < 0.1.

*Notes:* The augmented version of the Arellano-Bond GMM estimator is used in all specifications. Column 2 includes a placebo test where instead of using imported malaria incidence, I use imported non-malaria disease incidence. In column 3, the dependent variable is non-malaria disease incidence, while imported malaria incidence is calculated as is done in the baseline specification. In column 4, I do not weight movement observations to scale up to the full population of Senegal. In column 5, I scale the expected imported incidence and total incidence by the health post utilization of the region based on DHS data.

Table 3, column 4, uses an estimate of imported incidence without weighting the mobility data to be representative of the full population. This assumes that the only movement in the country is the movement in the Sonatel data, which would be an underestimate of movement and an upper bound for the coefficient estimate. Weighting the data represents an overestimate for the level of movement and a lower bound for the coefficient estimate. The effect should therefore lie between these upper and lower bounds of 2.1 and 1.7. Column 5 scales imported incidence and total incidence by health post utilization at the region level. The results are similar and not significantly different from the results in the main specification (column 1). The results remain statistically significant. The results from additional robustness checks are available in Appendix A.

There are three important limitations of the mobile phone data. First, I only use 12 months of mobility data and cannot control for annual patterns. Sonatel was unwilling to provide additional data at this time due to the resource and time costs. Nevertheless, they are part of an initiative called OPAL, which will provide additional access to limited data for researchers in the future, but this is not yet operational.

Second, the location estimates are based on cell tower location. For a tower located at the border of two districts, it could lead to misclassification of the origin. Although there is heterogeneity in malaria incidence in Senegal, there is very high spatial correlation in the incidence. Therefore, even if a person's origin is incorrectly attributed to the neighboring district, the incidence is likely similar in the two districts, so there should be limited measurement error in the person's contribution to an imported case. I defined health facility areas to minimize this potential ambiguity in location when focusing on the destination.

Third, the mobile phone data only include mobility within Senegal and cannot incorporate international migration. Immigrants from high-malaria countries as well as emigrants returning from high-malaria settings to visit family could impact malaria incidence. Detailed case data are available from Richard Toll district where each case was investigated and travel information was included on the infected individual based on survey data. Among 161 cases, the traveler was from outside Senegal in nine cases, or only 5.59 percent of the cases. The small impact of international migration likely arises because the only international border near the pre-elimination districts studied here is Mauritania, which has a lower incidence rate than northern Senegal of only 0.4 case per 1000.

## 5 Policy targeting strategies

I study effective allocation of resources to mitigate the negative externality of travel that was quantified in the previous section. I first describe some of the existing policies implemented in Senegal toward travelers. Then I conduct simulations to demonstrate the effectiveness of strategically targeted policies.

### 5.1 Malaria policies toward travelers

The nonprofit PATH has focused on reducing imported cases of malaria in the Richard Toll district. PATH has used volunteers in the community to alert health workers to the arrival of new travelers. Health workers track down these travelers, ask to test them using an RDT, and treat those who test positive. Based on 2015 data provided by the Richard Toll Health District Director, 3609 people were identified as travelers, and of these, 3386 were tested and 10 tested positive for malaria. In 2015, there were a total of 186 imported cases; therefore, this strategy was only able to detect 5 percent of the imported cases.^28^

**Fig. 4:**
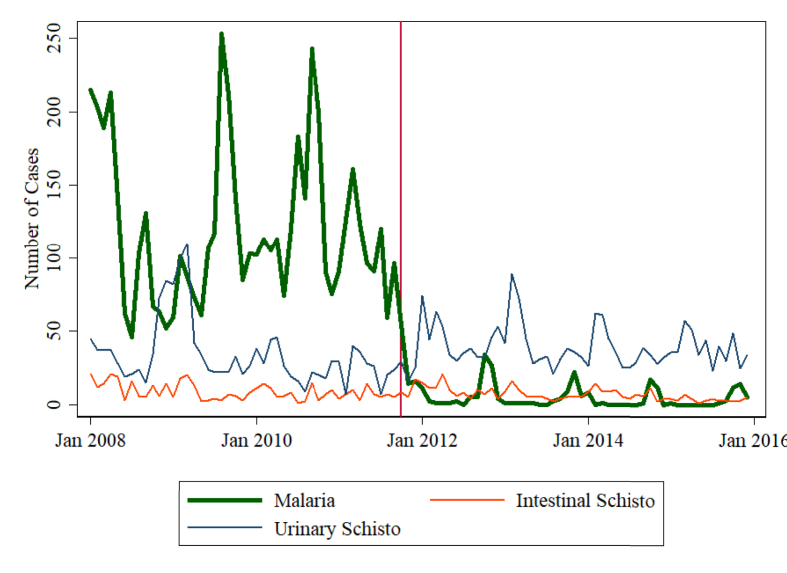
Effect on Malaria Cases of a Policy Targeting Migrant Workers at the Senegalese Sugar Company. *Notes*: The figure shows the number of cases of malaria and two types of schistosomiases seen at the health post of the Senegalese Sugar Company. The vertical line marks the timing of when a new policy was implemented by the company that tested every migrant worker for malaria and treated those who tested positive. Data were provided by the CSS.

A more systematic policy to target travelers was implemented by the Senegalese Sugar Company (CSS). The company hires more than 3000 migrant workers every year to help with the sugar harvest. Malaria was a large burden for the company, causing lower productivity, high absenteeism, and high spending on pharmaceuticals (Djibo and Ndiaye, 2013). In late 2011, the CSS implemented a new mandatory policy for all seasonal workers: testing every worker at the beginning of the season using an RDT, treating anyone testing positive, and providing workers and their families with bed nets and information. There was a drastic decrease in the number of cases after the implementation of this policy, with case numbers close to zero after the policy (Fig. 4). Data on two types of schistosomiasis among workers at the CSS show no drop in those diseases after late 2011, demonstrating that the drop in malaria cannot be attributed to an overall improvement in the health post. This mirrors the outcomes measured by Dillon et al. (2014), who find that a policy offering testing and malaria treatment for workers at a sugarcane plantation in Nigeria leads to a 10 percent increase in earnings due to increased labor supply and productivity.

A strategy to decrease malaria cases from travelers is proactive community treatment. The pilot of this intervention consisted of home care providers going door-to-door weekly to every household in a village, checking for individuals with symptoms. Compared with villages that did not receive the treatment, the odds of symptomatic malaria were 30 times lower in the intervention villages (Linn et al., 2015). Although it is effective, this type of program is costly and difficult to scale. Yet, it could be targeted to areas that receive travelers during the weeks when the most expected infections enter.

Mobile phones can also be used directly for targeting (see Lai et al. (2019) for a review of recent advances in mobile health technology). Mobile phones are always linked to a tower if they are turned on; therefore, the provider knows as soon as an individual changes towers. Users could, for example, opt into an information program that sends a targeted text message as soon as they change location from a high-malaria tower to a low-malaria tower. The message could recommend and provide an incentive to get tested at the closest clinic for free. Text messaging has been used by the Ministry of Health in Senegal for providing information on diabetes, and other studies have found that SMS technology can be effective in changing behavior (Senegal Ministry of Health, 2016; Aker and Ksoll, 2019; Ksoll et al., 2014; Fischer et al., 2016). Mobile phones could be used for more controversial policies such as targeting mandatory quarantine or contact tracing. Although contact tracing has been an important tool for epidemiologists for a long time (Eames and Keeling, 2003), the recent COVID-19 pandemic has highlighted the potential role of mobile phones. This can range from using the telecom records of diagnosed individuals to trace their movement, to applications that use bluetooth to establish individuals who came in contact with an infected person. There are significant privacy considerations and ethical concerns with these uses of mobile data, as is discussed by Raskar et al. (2020) and Cho et al. (2020). The severity and risk of the disease will affect the strategies that are considered appropriate.

### 5.2 Targeting simulations

The policies in the previous section serve as examples, but I focus the simulations on how a policy should be targeted, rather than the particular policy. If every traveler coming into the five pre-elimination districts that are the focus here were tested, it would mean testing 6,956,197 travelers in 2013 based on the scaled mobile phone data. This is infeasible from a cost and logistics perspective; therefore, only a subset of travelers should be targeted.

I consider two targeting strategies: (1) target certain destination areas for travelers or (2) target travelers from specific origin areas. For both cases, there are two sets of costs: (1) fixed costs, which consist of training community health workers in investigation of travel cases, and (2) variable costs, which are the costs associated with investigating and treating travelers.^29^ I calculate the fixed and variable costs based on the 2015 Malaria Operational Plan for Senegal, which allocates $400,000 for this type of case investigation and targeting.^30^ For all strategies, I assume that 94 percent of those who are targeted agree to be tested.^31^

The first type of targeting focuses on destination areas. Resources to target travelers can be distributed across all health facilities or concentrated in a smaller set of facilities in certain areas and months. Without cell phone data, policy makers can use information from the previous year to identify the health facility areas/months with the highest levels of malaria. The cell phone data allow for more effective targeting. The piece of information currently not available to policy makers is the number of travelers entering an area. The cell phone data provide this information and, combined with the incidence from 2012, make it possible to estimate which health facility areas are most at risk from travelers in certain months. This can be especially effective because travel choices are not random. There are often high movement corridors between certain communities in a country. Therefore, it is likely that there are pockets that will be more affected by travelers coming from high-malaria areas. Limited resources can be spent more effectively training health professionals and tracking travelers only in these areas.

**Fig. 5:**
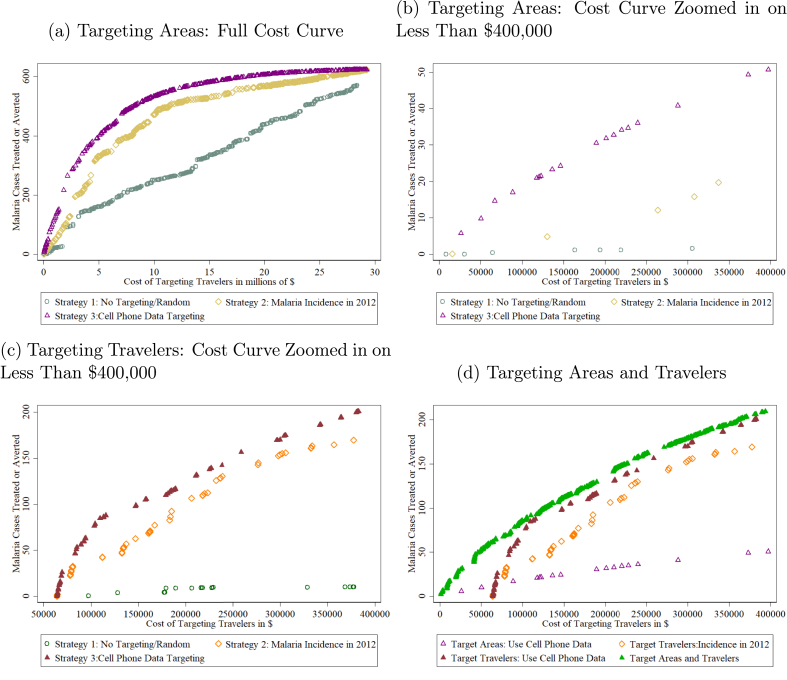
Targeting Policies: Cost and Benefit under Different Strategies. *Notes*: The panels show different strategies for targeting policies. In panels a and b, each symbol represents a health facility area-month. Targeting a health facility area-month means targeting all travelers entering that health facility area in that month. The strategies lay out which health facility area-months are targeted first. In panel c, each symbol represents a district-month. Targeting a district-month means targeting all travelers entering the five low-malaria districts from that district in that month. In panel d, a scenario where travelers from a particular district to a particular health facility area are targeted in a specific month is compared with previous scenarios of targeting particular health facility areas, targeting travelers from particular districts, and the scenario if no cell phone data are available of targeting travelers based on the incidence of districts in the previous year. The cost is calculated based on a variable cost of $2.85 per traveler and a fixed cost of $63,928 split proportionally between health facility areas based on population and number of facilities. The benefit is based on the parameters of the model to calculate the number of primary and secondary cases generated by travelers from each district in each month and summed for all travelers in a given health facility area-month. It is assumed that only 94 percent of those targeted are successfully tested.

Fig. 5, panel a, demonstrates the full cost curves, from a strategy where only one health district area-month is treated, to a strategy where all health district area-months are treated. The benefit at each point is calculated based on the earlier model to calculate the total primary and secondary cases attributed to travelers. Depending on the resources available to the government, it is possible to determine for any given budget how many cases would be treated or averted with each targeting strategy. For reference, the figure also includes a strategy of no targeting, where health facility areas are randomly chosen in random months. The targeted strategy based on telecom information is consistently the most cost-effective. Across the whole distribution, the cell phone data-based targeting policy performs 11.15 percent better on average compared with the next best policy of using information on incidence from the previous year. Zooming in on the part of the distribution up to $400,000, Senegal's budget for this type of program, the cell phone data targeted policy performs over 300 percent better on average (Fig. 5, panel b).

The second type of targeting focuses on travelers from particular origin areas in a given month. This is more difficult to implement because it requires the ability to identify travelers coming from specific areas, but it has the potential to be more cost-effective since resources are focused on the highest risk travelers. If a district-month is targeted, then every person traveling from that district to the five pre-elimination districts in that month would be targeted with the policy.^32^ I compare again with a strategy where the government uses monthly malaria incidence in the prior year to decide which travelers to target first and include the strategy of no targeting for reference. The cost starts at $63,928, which is the total fixed cost across all the pre-elimination health facility areas. Telecom data-based targeting is 27 percent more cost-effective on average than using incidence from the previous year, when focused on the available budget in Senegal of $400,000 (Fig. 5, panel c). Additionally, targeting specific travelers entering all five low-malaria districts (type 2) is 257 percent more effective than targeting all travelers who enter only specific health post catchment areas in those five districts (type 1), using the cell phone data for both.

I combine the two targeting strategies to target particular travelers going to particular areas in certain months (Fig. 5, panel d).^33^ This curve is compared with the previous curves of only targeting travelers, only targeting areas, and only targeting travelers based on incidence in 2012. Targeting travelers and areas leads to 52 percent better performance on average compared with the non-cell phone data strategy when focusing on Senegal's budget for this activity. Using cell phone data for targeting travelers and areas compared with just targeting travelers is 19 percent more effective on average.

There are two important limitations in conducting this type of targeting. The first is related to the potential risk of targeting areas based on movement information from a previous year and with only one year of data, given that population movement patterns may change drastically from one year to the next. Other research that has used cell phone data for Namibia finds consistent short-term movement patterns across three years (Milusheva et al., 2017; Wesolowski et al., 2017). Additionally, if short-term movement matrices from cell phone data were made available to policy makers on an ongoing basis, it would be possible to adjust targeting in real-time as information becomes available.

The second limitation relates to representativeness and who may be missed through these targeting strategies (Blumenstock, 2018). The movement patterns of the lowest income people are likely missing from these data due to lack of a mobile phone. Therefore, targeting could miss marginalized areas that may experience importation of malaria from very low-income groups that are not captured in the movement patterns. If policy makers implemented mobile phone data targeting strategies, it could lead to marginalized pockets of malaria in the elimination zones due to lack of targeting the areas receiving the lowest income travelers. The surveillance systems at health facilities in elimination zones that track malaria cases that come to the facility can be used to identify outlier health facilities that do not experience a decrease in malaria after the targeting strategies are implemented.

So far, I have not discussed high malaria districts from where cases are imported. If malaria were reduced significantly in those districts, it would automatically reduce importation. The assumption is that while strategic targeting is done in the low-malaria zones, in the high-malaria districts, a package of interventions aimed at reducing the burden of the disease is maintained. This is in line with the WHO strategy for malaria elimination.

## 6 Conclusion

The paper quantifies the negative externality of population movement on disease incidence and the reversal of gains in elimination of malaria in Senegal, and it proposes a cost-effective targeting strategy. This type of study is made possible by (1) new big data collected by telecommunications companies, making the measurement of short-term movements possible and (2) the initiative taken by the Ministry of Health to collect surveillance data on a monthly level for each health post. The study finds that for each imported case of malaria per 1000 people, there are around 1.7 cases of malaria per 1000 reported at health posts in the pre-elimination districts. Using these findings, I calculate that implementing a policy in certain destination areas and targeting travelers from the most cost-effective districts during particular months results in the largest drop in cases (over 50 percent as many cases treated or averted as compared with the next best strategy). This would not be possible without cell phone data, which provide extremely granular information on the spatial movement of individuals across time so that we know how many travelers enter a specific area in a given month.

It is possible to implement this type of model for other infectious diseases such as influenza, Ebola, Zika, or COVID-19, with modifications to account for the specific epidemiology of each disease and the movement that is relevant. These diseases similarly require the detailed data on mobility that can be generated from mobile phones in combination with up-to-date case data. Incorporating these types of models into targeting policies could help countries lower the disease burden from existing infectious diseases and prevent epidemics of new diseases, leading to beneficial economic and social consequences.

## Figures and Tables

**Fig. A.1:**
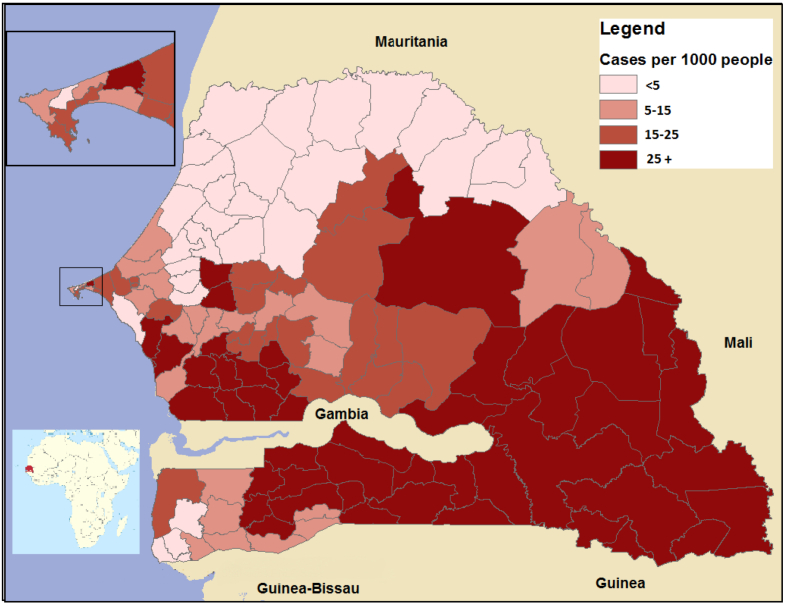
Annual Malaria Incidence in Senegal in 2013. *Notes*: Data come from tested and confirmed cases at the health district level compiled by the National Malaria Control Program.

**Fig. A.4:**
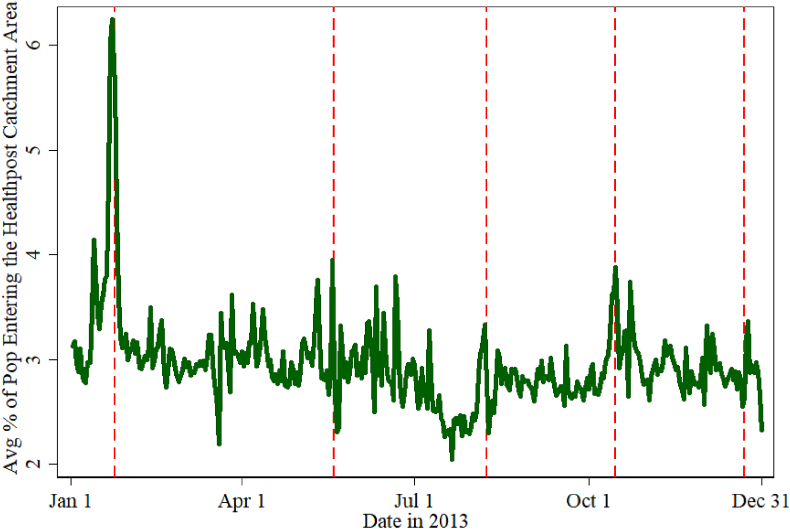
People Entering a Health Post Catchment Area as a Percent of the Population in the Area, Avg Across All Health Post Catchment Areas. *Notes*: Red dash lines represent some religious holidays and pilgrimages.

**Fig. A.5:**
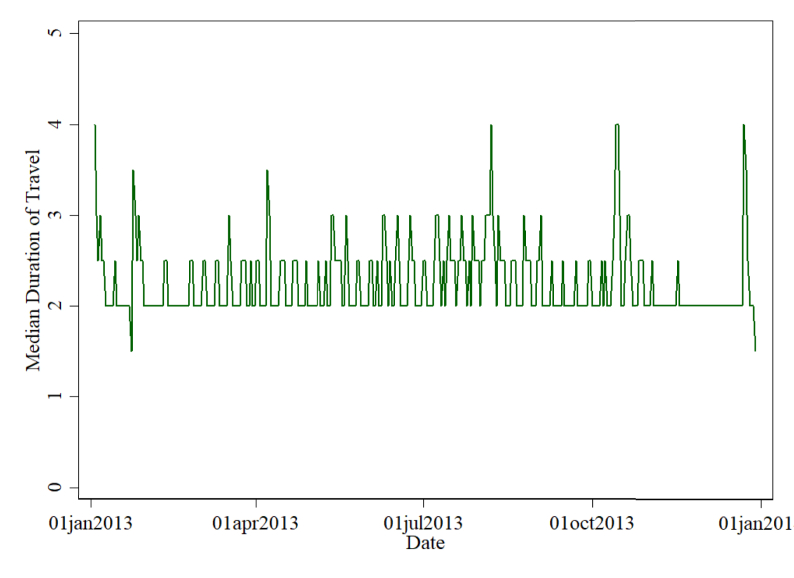
Median Duration of Trips, by Day of the Trip.

**Table Al:** Using Different Rainfall Specifications.

	(1)	(2)	(3)	(4)	(5)	(6)
	Baseline	No Rain Lags	Only 1 Month Lagged Rain	Only 2 Months Lagged Rain	Quadratic Functional Form of Rain	Logged Rain
Imported Incidence	1.094∗∗∗	1 313∗∗∗	1.338∗∗∗	1.203∗∗∗	1.087∗∗∗	1.284∗∗∗
	(0.451)	(0.505)	(0.497)	(0.446)	(0.487)	(0.542)
Lag Incidence	0.563∗∗∗	0.597∗∗∗	0.598∗∗∗	0.563∗∗∗	0.510∗∗∗	0.658∗∗∗
	(0.126)	(0.117)	(0.115)	(0.129)	(0.137)	(0.127)
Rain in cm	-0.000654	-0.00846			-0.000416	
	(0.00844)	(0.00855)			(0.0326)	
Lag Rain in cm	0.0269		0.0170		-0.0602	
	(0.0193)		(0.0173)		(0.0541)	
Lag 2 Rain in cm	0.0319∗∗			0.0228	-0.0395	
	(0.0169)			(0.0149)	(0.0498)	
(Rain in cm)^2^					-0.000200	
					(0.00179)	
Lag (Rain in cm)^2^					0.00429	
					(0.00316)	
Lag 2 (Rain in cm)^2^					0.00369	
					(0.00296)	
Log Rain in cm						-0.0117
						(0.0155)
Lag Log Rain in cm						-0.0232
						(0.0239)
Lag 2 Log Rain in cm						0.0224∗∗
						(0.0130)
Health Post x Month Obs	432	432	432	432	432	283
Month FE	Yes	Yes	Yes	Yes	Yes	Yes

Cluster bootstrap standard errors in parentheses.

∗∗∗ p < 0.01, ∗∗ p < 0.05. ∗ p < 0.1.

*Notes*: The augmented version of the Arellano-Bond GMM estimator is used in all specifications.

**Table A2:** Effect of Imported Malaria Incidence, Robust Standard Errors

	(1)	(2)	(3)	(4)
	Fixed Effects	Fixed Effects	Random Effects	Arellano Bond
Imported Incidence	1.230∗∗ (0.452)	1.175∗∗ (0.476)	0.864∗∗ (0.381)	1.094∗∗∗ (0.357)
Lag Incidence	0.330∗∗∗ (0.0509)		0.418∗∗∗ (0.0552)	0.563∗∗∗ (0.129)
Lag Imported Incidence		0.449∗ (0.231)		
Lag Non-Imported Incidence		0.340∗∗∗ (0.0571)		
Rain in cm	0.00639 (0.00934)	0.00592 (0.0102)	0.00449 (0.00853)	−0.000654 (0.00790)
Lag Rain in cm	0.0292 (0.0189)	0.0294 (0.0188)	0.0300∗ (0.0177)	0.0269 (0.0181)
Lag 2 Rain in cm	0.0381∗∗ (0.0158)	0.0372∗∗ (0.0155)	0.0382∗∗∗ (0.0141)	0.0319∗∗ (0.0156)
Month FE	Yes	Yes	Yes	Yes
Health Post x Month Obs	432	396	432	432
R-squared	0.509	0.512		

Robust standard errors in parentheses.

∗∗∗ p < 0.01, ∗∗ p < 0.05, ∗ p < 0.1.

*Notes:* Column 3 includes controls for health post area population density, a dummy for urban health post areas, and a dummy for non-border health post areas.

**Table A3:** Robustness Checks

	(1)	(2)	(3)	(4)	(5)	(6)	(7)
	Baseline	Spatial and Autocorrelation SE	Net Imported	Cases	Adjusted Pop	Scale by Susceptible Pop	Restrict Phone Data
Imported Incidence/Cases	1.094∗∗∗ (0.451)	1.230∗∗∗ (0.326)	1.217∗∗∗ (0.504)	1.077∗∗∗ (0.667)	1.070∗∗∗ (0.465)	1.091∗∗∗ (0.440)	1.495∗∗ (0.703)
Lag Incidence/Lag Cases	0.563∗∗∗ (0.126)	0.330∗∗∗ (0.0543)	0.561∗∗∗ (0.126)	0.684∗∗∗ (0.234)	0.619∗∗∗ (0.0808)	0.625∗∗∗ (0.0944)	0.575∗∗∗ (0.126)
Rain in cm	−0.000654 (0.00844)	0.00640 (0.0086)	−0.000852 (0.00843)	−0.273 (0.220)	0.000636 (0.00866)	−0.000471 (0.00859)	−0.00140 (0.00843)
Lag Rain in cm	0.0269 (0.0193)	0.0292∗ (0.0167)	0.0258 (0.0192)	0.188 (0.742)	0.0243 (0.0188)	0.0268 (0.0194)	0.0262 (0.0194)
Lag 2 Rain in cm	0.0319∗∗ (0.0169)	0.0380∗∗ (0.0149)	0.0318∗∗ (0.0169)	0.860∗ (0.591)	0.0322∗∗ (0.0159)	0.0306∗∗ (0.0161)	0.0315∗∗ (0.0166)
Month FE	Yes	Yes	Yes	Yes	Yes	Yes	Yes
Health Post x Month Obs	432	432	432	432	432	432	432

Cluster bootstrap standard errors in parentheses.

∗∗∗ p < 0.01, ∗∗ p < 0.05, ∗ p < 0.1.

*Notes:* The augmented version of the Arellano-Bond GMM estimator is used in all specifications. In column 2, I use Conley standard errors with a 30-km cutoff for the spatial correlation and two lags for the autocorrelation. These account for spatial autocorrelation and serial autocorrelation over time, but do not cluster at the health post catchment area level. In column 3, I calculate net imported incidence by subtracting the expected cases, leaving a health facility area divided by the population of the area. In column 4, I use cases rather than cases per 1000 people. In column 5, the population of the health post catchment area is adjusted monthly by the number of people entering and exiting based on the mobile phone data scaled by a factor of.7 to account for the population not represented by these data. In column 6, lag incidence is scaled by the percentage of the population that is susceptible to malaria. In column 7, movements are restricted to those where the SIM card had a call or a text within 14 days of when the movement is counted.

**Fig. B.1:**
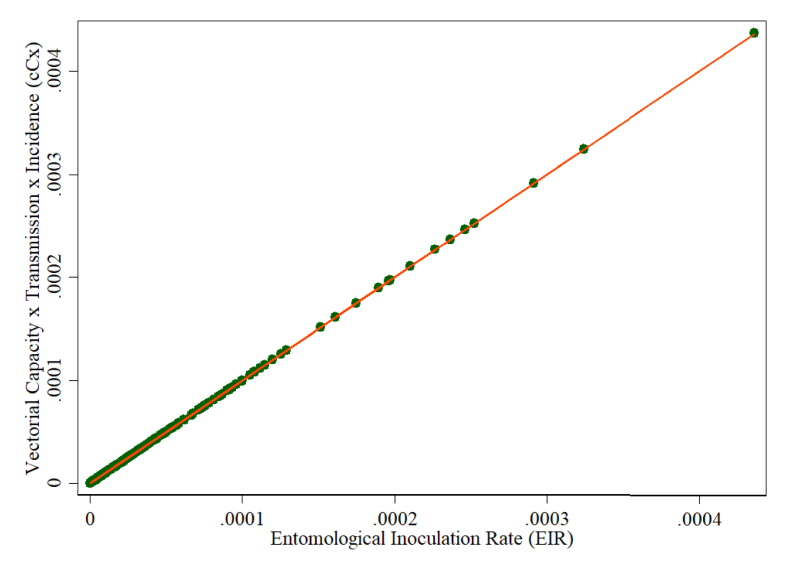
Testing the Approximation of the Entomological Innoculation Rate in Assumption 4. *Notes*: For each health post area in each month, the entomological inoculation rate is calculated as the vectorial capacity times transmission to mosquitoes times incidence (cCx). Based on assumption 4, in low-malaria districts it should be possible to approximate EIR with cCx. In the scatter plot, this means the points should be along the 45-degree line, which is shown in red. The graph shows that for the districts analyzed here, the assumption holds. The EIR and cCx were estimated using values from the literature for the various biological malaria parameters. Based on Gething et al. (2011), average transmission from infected humans to mosquitoes, c, is 0.161. The incubation period, τ, is nine days (Killeen et al., 2006) and the bites on humans per mosquito, a, is 0.3 (Ruktanonchai et al., 2016). Average mosquito lifespan of 12.5 is used.

## Appendices

### Appendix B Model of Malaria

Due to the two-host system, malaria is modeled using two differential equations to describe the dynamics of infected humans and infected mosquitoes. These were first modeled by Ross (1910) and then expanded by Macdonald et al. (1957). The model used in this paper is a Ross-Macdonald type model based on models used in Smith and Ellis McKenzie (2004), Cosner et al. (2009), and Torres-Sorando and Rodriguez (1997). There are two state variables and the model is usually expressed in continuous time, although here I present it in discrete time. The state variables are *y*_*it*_, the fraction of mosquitoes infected in location i at time t, and *x*_*it*_, the fraction of humans infected in location i at time t.

In the model extension here, which includes the impact of population movement, cases can be generated locally in location i or imported from any other location j into i, $I_{i}$. As modeled by Torres-Sorando and Rodriguez (1997), imported cases enter the model linearly. Local cases in location i are generated based on the susceptible population and several biological parameters. The susceptible population, *S*_*it*−*w*_, is the fraction of the population that is susceptible to malaria at time *t* − *w*, where *w* is the total parasite incubation period. The other parameters are the transmission efficiencies from infected mosquitoes to humans and humans to mosquitoes, *b*_*it*−*w*_ and *c*_*it*_, the number of bites on humans per mosquito, *a*_*it*−*w*_, and the ratio of mosquitoes to humans, *m*_*it*−*w*_. The change in the number of infected humans and mosquitoes is described by:

(B.1) $$\begin{array}{ccc} y_{it}-y_{it-w} & = & a_{it-w}c_{it-w}x_{it-w}\left(e^{-\mu_{it-w}\tau_{it-w}}-y_{it-w}\right)-\mu_{it-w}y_{it-w} \end{array}$$

(B.2) $$\begin{array}{ccc} x_{it}-x_{it-w} & = & m_{it-w}a_{it-w}b_{it-w}y_{it-w}S_{it-w}+I_{it}-r_{i}x_{it-w} \end{array}$$

where *μ*_*it*_ is the mortality rate of mosquitoes and *τ*_*it*_ is the incubation period from the time a mosquito becomes infected until it is infectious. The parameter *r*_*i*_ is the recovery rate of humans. I make four assumptions arising from the epidemiology literature based on modeling the system in a low-malaria setting:

1. The total parasite incubation time is one month based on the malaria cycle.
2. The mosquito population is at the steady state, since mosquito populations have a relatively rapid turnover.^34^
3. All malaria cases in month t are treated immediately and recover in month t.^35^
4. Based on Smith and Ellis McKenzie (2004), when the proportion of infected humans is small, the number of infectious bites received per day by a human (known as the entomological inoculation rate, taking the form $EIR_{it}=\frac{m_{it}a_{it}^{2}c_{it}e^{-\mu_{it}\tau_{it}}x_{it}}{\mu_{it}+a_{it}c_{it}x_{it}}$) can be approximated by *c*_*it*_*C*_*it*_*x*_*it*_, where *C*_*it*_ is the expected number of humans infected per infected human per day, assuming perfect transmission efficiency (*b*_*it*_ = *c*_*it*_ = 1), known as the vectorial capacity. The assumption applies because the analysis is conducted in a low-malaria setting. Figure B.1 provides evidence that the assumption holds for these data.

Based on assumption 1, I set *w* = 1. Based on assumption 2, I solve equation B.1 for the quasi-equilibrium proportion of infectious mosquitoes as has been done in Smith and Ellis McKenzie (2004) and Ruktanonchai et al. (2016):

(B.3) $$y_{it-1}=\frac{a_{it-1}c_{it-1}x_{it-1}e^{-\mu_{it-1}\tau_{it-1}}}{\mu_{it-1}+a_{it-1}c_{it-1}x_{it-1}}$$

Assumption 3 implies the recovery rate, *r*_*i*_, is equal to one, since all infected individuals recover within the same month. This allows me to focus on new cases of malaria in month t. Since immunity does not develop in low-incidence areas, anyone who is not currently infected is susceptible to the disease, which implies that the susceptible population is 1 if everyone recovers. Rewriting equation B.2 to incorporate the implications of assumptions 1–3:

(B.4) $$x_{it}-x_{it-1}=\frac{a_{it-1}^{2}b_{it-1}c_{it-1}m_{it-1}e^{-\mu_{it-1}\tau_{it-1}}x_{it-1}}{\mu_{it-1}+a_{it-1}c_{it-1}x_{it-1}}+I_{it}-x_{it-1}$$

Based on assumption 4, equation B.4 can be rewritten as:

(B.5) $$\begin{array}{ccc} x_{it} & = & b_{it-1}EIR_{it-1}+I_{it}\\ & = & b_{it-1}c_{it-1}C_{it-1}x_{it-1}+I_{it} \end{array}$$

Assumption 4 is important because by rewriting *EIR*_*it*−1_ as *c*_*it*−1_*C*_*it*−1_*x*_*it*−1_, I explicitly incorporate the impact of the incidence last month, *x*_*it*−1_, on the incidence in the current month using a linear functional form, which helps me approximately estimate the secondary cases generated this month by cases last month. It is then possible to estimate this model using OLS as described in the main text of the paper.

I can use the existing epidemiological literature to predict the size of the *b*_*it*−1_*c*_*it*−1_*C*_*it*−1_ term, which corresponds to the *β*_1_ coefficient in the main paper that measures the number of new cases generated this month due to incidence last month. Based on Vercruysse et al. (1983), the average daily vectorial capacity in Senegal, C, is 1.13. Based on Gething et al. (2011), average transmission from infected humans to mosquitoes, c, is 0.161. The transmission from mosquitoes to humans, b, is 0.05 based on the linear model in Smith et al., (2010). This gives an average daily value of 0.0091, or a monthly value of 0.273 for *bcC*. This is compared with the estimated coefficient from the regression model in the paper.

The use of incidence data separates this paper from some of the previous work that relies on endemicity data. The endemicity data are gathered from parasite rate surveys in which a random subsample of the population is tested for malaria parasites. When the malaria prevalence is very low, the likelihood of having a positive case becomes very small. Therefore, when focusing on a low-malaria setting to understand the impact of mobility, incidence is a more reliable measure (Alegana et al., 2013; Cohen et al., 2013).

## Footnotes

1 Data as of August 16, 2020.

2 See Currie and Vogl (2013) and Apouey et al. (2018) for summaries of the literature.

3 In Senegal, census data only include long-term migration statistics every 10 years. Other surveys ask about commuting, but they are usually only for one point in time and one area.

4 The long incubation period for malaria allows people to travel without knowing they are infected.

5 This paper does not focus on the type of targeting, but examples can include information campaigns targeted to travelers via mobile phone and strategically setting up testing sites.

6 The data they use come from the original D4D Challenge, which only provided a sample of data with a degraded spatial resolution at the arrondissement level. A few researchers that took part in this challenge were given access to the full raw and anonymized telecom data for Senegal at the cell tower level, and this is what is used for the current paper.

7 The incubation period can vary, but two sites in Senegal had an average of nine days.

8 Unlike other malaria parasites, *P. falciparum* does not have the potential to lie dormant for months.

9 Details on malaria transmission can be found in Doolan et al. (2009), Smith and Ellis McKenzie (2004), Killeen et al. (2006), and Wiser (2010).

10 The average radius of travel for mosquitoes that carry the malaria parasite in Senegal is only 1–2 km (Russell and Santiago, 1934; Thomas et al., 2013).

11 Figure A.1 in Appendix A shows a map of annual malaria incidence by district in 2013.

12 The data come from the PNLP and PATH, a nonprofit organization working with the PNLP to fight malaria in Senegal through its Malaria Control and Elimination Partnership in Africa.

13 Rainfall data are from the Climate Prediction Center (2016) Rainfall Estimator for Africa.

14 This is comparable to the rest of Sub-Saharan Africa, where 50–80 percent of rural households were estimated to have at least one migrant (Deshingkar and Grimm, 2005).

15 At this time it was not possible to obtain more recent data or data from other providers.

16 Table A3 in Appendix A provides a robustness check removing observations with more than 14 days in a row missing.

17 For diseases where any contact may lead to further spread of the disease, one important limitation is the frequency of mobile phone use that limits the number of movements measured. GPS tracking information from mobile apps or signaling data from mobile phones, could generate higher resolution information, but many countries have limited availability of such data.

18 Movements within a district between health post catchment areas are not counted.

19 The survey does not contain data on men.

20 Population is based on the known population of each health facility catchment area. Table A3 in Appendix A includes a robustness check where the population is adjusted monthly using the telecom data.

21 See Figure A.5 in Appendix A for median duration by day.

23 Additional functional forms of rainfall were tested but did not significantly change the analysis. Table A1 in Appendix A includes results with these different specifications.

24 I include a robustness check with spatial and panel autocorrelated standard errors in Table A3 in Appendix A. I also include results with cluster robust standard errors in Table A2 in Appendix A.

25 See Appendix B for detailed calculations.

26 I would not expect a persistent effect of imported cases two months out because the malaria season when the necessary mosquito vector is present in these areas is very short, lasting only around three months; therefore, there is not enough time for tertiary cases to develop due to the month-long incubation period.

27 The average number of travelers was calculated based on the total number of travelers and unique origin-destination pairs in a given month.

28 The data on imported cases are based on surveys of positive malaria cases conducted in Richard Toll.

29 For simplicity, I assume the variable cost scales proportionally with the number of travelers.

30 The $400,000 is for the 19 districts with incidence less than 5/1000. I split this funding proportional to district population to assign an amount to the five districts that are part of the simulation. This leads to $127,850 dedicated to the five low-malaria districts studied here. I assume an even split between fixed and variable costs. To calculate the variable cost per person, I use the information available from Richard Toll, where 3609 people were targeted in 2015. Variable funding for that district of $10,286 leads to a per person cost of $2.85.

31 This is based on data from Richard Toll, where 3386 of 3609 targeted travelers agreed to be tested.

32 The cost is calculated for each district-month based on the number of travelers and the variable cost and the benefit is calculated as the impact of the imported cases using incidence from the previous year.

33 The fixed cost of an area is added when the first set of travelers in a month is treated in that area.

## Footnotes

22 I use detailed knowledge of the timing from the mobile phone data to factor in how many of the 15 days were in month t and how many in month *t* − 1 and use the incidence in months *x*_*jt*_ and *x*_*jt*−1_ to determine the probability the person is infected.
